# Determining Regional Differences in Barriers to Accessing Health Care Among Farmworkers Using the National Agricultural Workers Survey

**DOI:** 10.1007/s10903-022-01406-9

**Published:** 2022-11-01

**Authors:** Sheila Soto, Aaron Meck Yoder, Benjamin Aceves, Tomas Nuño, Refugio Sepulveda, Cecilia Ballesteros Rosales

**Affiliations:** 1 Division of Public Health Practice & Translational Research, Mel and Enid Zuckerman, College of Public Health, University of Arizona, Phoenix, AZ USA; 2 Department of Epidemiology & Biostatistics, Mel and Enid Zuckerman College of Public Health, University of Arizona, Tucson, AZ USA; 3 School of Public Health, San Diego State University, San Diego, CA USA

**Keywords:** Farmworker, NAWs, Access, Health services

## Abstract

Farmworkers are an essential workforce in the U.S. We assessed the regions in the National Agricultural Workers Survey on the difficulty of accessing health care among farmworkers in the U.S. The study included 9577 farmworkers. Farmworkers in all regions were more likely to report having difficulty accessing health care because it was too expensive. The overall odds ratio for difficulty accessing health care was lower in the MW after adjusting. Farmworkers employed in the SE had greater difficulty accessing health care because of language barriers. Farmworkers employed in CA had difficulty accessing health care in the U.S. because it was too expensive or far away. Results follow previous studies on barriers to access health care among the farmworker population. Understanding regional disparities in the presence of barriers to accessing health care among farmworkers is an essential step to improving equitable health care access in the U.S.

## Introduction

The farmworker population residing in the U.S. is an essential workforce critical to our economy and food system productivity [1, 2]. Presently, there are nearly four million farmworkers in the U.S., with estimates up to 3 million workers classified as migrant or seasonal [1, 3]. Even with exposures to occupational hazards and injury rates as high as 12.5%, farmworkers lack occupational health standards and fair labor laws [4, 5]. Farmworkers face low socioeconomic factors, including high rates of poverty, low levels of educational attainment, and inadequate housing conditions [6, 7]. Despite being an essential labor force, farmworkers encounter barriers to accessing health care in the U.S.

Multiple studies have reported barriers to accessing health care among farmworkers. Many farmworkers live and work in rural areas, which present unique challenges in reaching care, such as difficulty attaining transportation to and from appointments [8–10]. Farmworkers, specifically those working in certain areas or crops are often immigrants, or have family members who are immigrants, and have reported fear of immigration enforcement as a barrier to accessing health care [8–10]. Similarly, many farmworkers with limited English proficiency have reported language barriers resulting in both access and quality of care issues [8–10]. The increasing cost of health care services is a national problem, but with a large portion of farmworkers living below the poverty line—health care costs have become an astronomical barrier in accessing services [8–10].

Barriers to health care access have lasting adverse effects on farmworker health outcomes. For instance, farmworkers lacking critical primary care, which limits primary and secondary prevention efforts, have an increased risk of suffering from an undiagnosed chronic disease [8, 11]. This in turn hinders access to early treatment and management to prevent further complications [8, 11, 12]. In addition, populations that have difficulty accessing primary care tend to rely on urgent or emergency care, which is both economically impractical and dangerous for patients [10].

Improving health outcomes for farmworkers in the U.S. requires a broad approach, including policy changes to labor laws and expanding access to health care for all farmworkers. Assessing and understanding disparities in farmworkers’ access to health care services can improve the allocation and development of resources to ensure farmworkers do not face barriers when accessing health care. A patchwork of local, state, and federal laws currently attempts to ensure the agricultural workforce’s health and safety; thus, discrepancies in barriers to accessing health care may exist depending on geographic location [13]. Also, the type of crop work, migratory patterns, season, citizenship, or visa status of farmworkers may be vastly different per geographical region of employment [14, 15] The objective of this study is to determine regional differences in barriers farmworkers face when accessing health care in the U.S. using the National Agriculture Workers Survey (NAWS).

## METHODS

### Study Design

The use of de-identified secondary publicly available data exempts this research from approval by the Institutional Review Board or Ethics Review Committee. We verified that all data was de-identified upon extraction of the datasets from the NAWS 2013–2016 before beginning the statistical analysis [16]. The NAWS is a national survey conducted by the U.S. Department of Labor (DOL) designed to provide a representative sample of hired farmworkers. The DOL, Employment and Training Administration (ETA) makes formal request for approval to the Office of Management and Budget (OMB) for the methodology of the NAWS. The NAWS uses an intricate, multistage survey design to account for seasonal and regional farmworker employment changes. There are seven layers of sampling for the primary study including crop cycle, region, farm labor area, county, zip code, employer, and crop workers. NAWS samples 3 times a year in 12 geographic regions and randomly selects employers within randomly sampled Farm Labor Areas (FLA) resulting in 36-time-by-space strata. The selection of cycles, regions, and farm labor-areas is dependent on the amount of farm labor in a region during the collection cycle [17]. The selection of counties, ZIP codes, and employers is determined by the farm expenditures in FLA and information from employers from the Quarterly Census of Employment and Wages and commercial lists of employers or other related data [16]. Between 1500 and 3600 farmworkers are randomly selected for interviews at their worksite during breaks or before or after work on an annual basis [17]. The NAWS excludes H2A Visa holders and farmworkers who carry out farm-related tasks not related to crop work such as those who exclusively work with livestock or dairy [16]. Most workers have a place of birth in Mexico (68.5%), U.S. (26.0%), Central America (5.0%), or other (1.0%) [18]. Length of time in the U.S. in years is not captured by the NAWS, instead variables of migrant type recorded including settled (did not migrate; 82.5%), shuttle migrant (10.0%), follow-the-crop migrant (5.0%), or foreign-born newcomer (3.0%) [18].

### Exposure

**Fig. 1:**
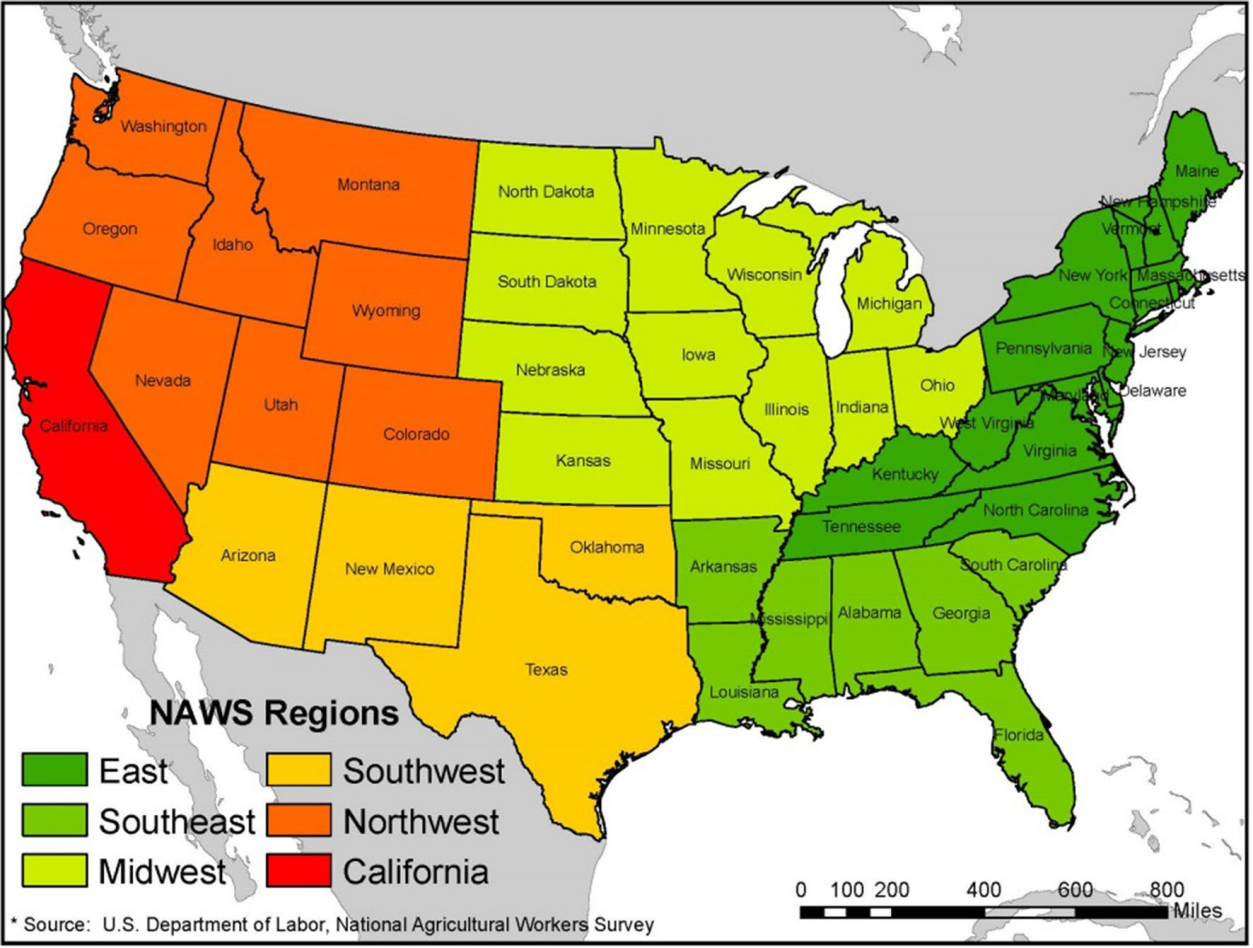
Geographical regions where farmworkers were selected to participate in the NAWS

This study assessed the effect of employment in the six U.S. regions on the difficulty of accessing health care among farmworkers in the U.S. We analyzed the regions using the NAWS predetermined subgroups of Northeast (NE), Southeast (SE), Midwest (MW), Southwest (SW), Northwest (NW), and California (CA) all on the 17 USDA-designated regions (Fig. 1). Due to the size and number of farmworkers in California a separate subgroup was created during the primary study for the state to not heavily impact if included in the surrounding regions. We assessed by regions to have an illustrative sample of the various types of agricultural crops and work seasons throughout the U.S. We calculated descriptive statistics for farmworkers in each region and CA, as the reference region, in all models.

### Measures

All variables in this study used during secondary data analysis were self-reported during interviews conducted during the NAWS primary study. The outcomes are the barriers that make accessing health care services difficult for farmworkers. We focused on three barriers (cost, language, and transportation). Each outcome was a binary response (Yes or No) and was assessed in the NAWS through the question: “When you want to get health care in the U.S. what are the main difficulties you face?” with the three options: “no transportation, too far away”, “do not speak my language”, and “too expensive” given separately.

We calculated descriptive statistics for age, gender, hourly wage, health insurance status, the origin of birth, and education level, gender (male or female), health insurance status (has health insurance, does not, or does not know), and origin of birth (U.S. or other) are categorical variables and include unweighted counts and percentages. The descriptive statistics, age, hourly wage, and education level are continuous variables, and the NAWS public dataset help determine the mean and standard error, the survey weights in the NAWS public dataset.

### Statistical Methods

First, we use a separate logistic regression model for each outcome, which adjusted for the NAWS survey weights to determine the odds of each outcome by region with California as the reference group. An adjusted odds ratio (OR) is an odds ratio that controls for other predictor variables. Then, three more logistic regression models adjusted for NAWS survey weights and hourly wage as a continuous response: age (< 30, 30–44, 45–59, 60 ≤ years old); gender (male, female); and health insurance status (insured, uninsured, or does not know), to determine the adjusted OR for each region. Age was categorized to meet the assumptions of logistic regression.

We performed a sensitivity analysis using imputed values for missing outcome and covariate data, using hot deck imputation for complex surveys [19]. After imputation, a logistic regression model for each outcome was fit to the data, adjusted for survey weights and previously mentioned covariate using SAS software version 9.4.16.

## Results

### Descriptive Statistics

The study included 9577 farmworkers with 1229 in the NE region, 1280 in the SE region, 1076 in the MW region, 727 in the SW region, 1470 in the NW region, and 3791 in CA. Descriptive statistics for age, gender, hourly wage, health insurance status, origin of birth, and education level stratified by region of employment are in Table 1. Overall, the mean age was similar for farmworkers in each region (lowest 36.3 in the NW and highest 41.9 in the SW). Approximately three-quarters of farmworkers in each region were male. The mean hourly wage was lowest in the SW region at $9.17 and highest among farmworkers in the MW at $11.25. About half (46.2%) of the farmworkers in the MW reported having health insurance, while only 25.8%, 33.4%, and 33.8% of farmworkers in the SE, NW, and SW regions had health insurance. Nearly half (44.9%) of farmworkers in the MW were born in the U.S., while only 8.4%, 18.7%, and 26% of farmworkers in CA, NW, and SW were born in the U.S., respectively. The mean education level was 10.5 years among farmworkers in the MW, while farmworkers in other regions averaged between 7.6 and 9.0 years of education.

**Table 1:** Descriptive statistics, farmworkers interviewed in the NAWS 2013–2016 (n = 9577)

	NE (n = 1229)	SE (n = 1280)	MW (n = 1076)	SW (n = 727)	NW (n = 1470)	CA (n = 3791)
Age, mean (SndEr)	37.3 (0.8)	37.1 (0.9)	40.2 (1.1)	41.9 (1.0)	36.3 (0.7)	38.6 (0.5)
Male gender, n (%)	954 (77.6%)	887 (69.3%)	799 (74.3%)	603 (82.9%)	1,137 (77.3%)	3,016 (79.6%)
Hourly wage, mean (SndEr)	$10.10 (0.2)	$9.61 (0.1)	$11.25 (0.2)	$9.17 (0.1)	$11.00 (0.3)	$10.49 (0.1)
Has health insurance, n (%)	411 (33.4%)	330 (25.8%)	497 (46.2%)	246 (33.8%)	518 (35.2%)	1,716 (45.3%)
Born in the U.S., n (%)	398 (32.4%)	401 (31.3%)	483 (44.9%)	189 (26.0%)	275 (18.7%)	317 (8.4%)
Education level, mean (SndEr)	9.0 (0.3)	8.5 (0.2)	10.5 (0.3)	8.0 (0.3)	8.1 (0.2)	7.6 (0.1)

Mean and standard error calculated using NAWS weights to adjust for sample design

*NE* Northeast, *SE* Southeast, *MW* Midwest, *SW* Southwest, *NW* Northwest, *CA* California

### Primary Outcomes

Farmworkers in all regions were more likely to report having difficulty accessing health care in the U.S. because it was too expensive (between 20.1 and 36.1%) compared to language or transportation problems (0.9–3.9 and 0.6–1.9%, respectively). The total count of farmworkers in all regions who reported having difficulty accessing health care because of expense was 2568, compared to 163 who reported having difficulty because of language barriers and 85 who reported having difficulty because of transportation or services were too far away.

The odds of farmworkers having difficulty accessing health care because it was too expensive for each region compared to CA can be found in Table 2. After adjusting for survey weights, age, gender, income, and health insurance status, the odds of reporting difficulty accessing care because of cost were lower in regions other than CA, and the results were significant in all regions except for in the SE region where the OR was 0.92 with 95% CI (0.79, 1.08).

**Table 2:** Association between regions and having difficulty accessing health care because it is too expensive, NAWS 2013–2016 (n = 9577)

	Count (%)^a^	OR unadjusted model (95% CI)	OR adjusted model^b^ (95% CI)	OR sensitivity analysis^c^ (95% CI)
NE	291 (23.8%)	0.88 (0.75, 1.02)	0.72 (0.61, 0.84)	0.72 (0.62, 0.85)
SE	459 (36.1%)	1.21 (1.04, 1.40)	0.92 (0.79, 1.08)	0.92 (0.79, 1.07)
MW	215 (20.1%)	0.66 (0.56, 0.77)	0.69 (0.58, 0.82)	0.68 (0.57, 0.80)
SW	183 (25.3%)	0.87 (0.72, 1.05)	0.70 (0.58, 0.86)	0.72 (0.59, 0.87)
NW	374 (25.5%)	0.95 (0.83, 1.09)	0.73 (0.63, 0.85)	0.75 (0.64, 0.87)
CA	1,046 (27.8%)	Reference	Reference	Reference

*NE* Northeast, *SE* Southeast, *MW* Midwest, *SW* Southwest, *NW* Northwest, *CA* California

^a^53 observations had missing outcome data

^b^Adjusted for age, gender, income, and health insurance status

^c^Odds ratio for adjusted model with imputed data for missing values among covariates

Results for farmworkers having difficulty accessing health care because providers do not speak farmworkers’ language are in Table 3. The adjusted odds that farmworkers in the NE and SE region reported having difficulty accessing health care because of language barriers was 1.66 (95% CI 0.98, 2.79) and 3.38 (95% CI 2.19, 5.22), respectively. Adjusted odds were lower in the MW but higher in all other regions than CA.

**Table 3:** Association between regions and having difficulty accessing health care because providers do not speak farmworker’s language, NAWS 2013–2016 (n = 9577)

	Count (%)^a^	OR unadjusted model (95% CI)	OR adjusted model^b^ (95% CI)	OR sensitivity analysis^c^ (95% CI)
NE	23 (1.9%)	1.95 (1.17, 3.25)	1.66 (0.98, 2.79)	1.63 (0.97, 2.73)
SE	49 (3.9%)	4.45 (2.91, 6.79)	3.38 (2.19, 5.22)	3.31 (2.16, 5.10)
MW	10 (0.9%)	0.68 (0.32, 1.43)	0.91 (0.43, 1.93)	0.85 (0.40, 1.80)
SW	12 (1.7%)	1.52 (0.78, 2.98)	1.31 (0.66, 2.61)	1.29 (0.64, 2.55)
NW	20 (1.4%)	1.25 (0.71, 2.18)	1.16 (0.65, 2.04)	1.12 (0.64, 1.97)
CA	49 (1.3%)	Reference	Reference	Reference

*NE* Northeast, *SE* Southeast, *MW* Midwest, *SW* Southwest, *NW* Northwest, *CA* California

^a^55 observations had missing outcome data

^b^Adjusted for age, sex, income, and health insurance status

^c^Odds ratio for adjusted model with imputed data for missing values among covariates

Table 4 shows the estimates for farmworkers’ difficulty accessing health care because it is too far away or lacks transportation. The adjusted odds were higher in CA than in all regions except for the SW, where it was 1.02 (95% CI 0.50, 2.08), compared to CA. The adjusted OR was significantly lower in the NE, MW, and NW.

**Table 4:** Association between regions and having difficulty accessing health care because it is too far away or farmworkers do not have transportation, NAWS 2013–2016 (n = 9577)

	Count (%)^a^	OR unadjusted model (95% CI)	OR adjusted model^b^ (95% CI)	OR sensitivity analysis^c^ (95% CI)
NE	7 (0.6%)	0.43 (0.19, 1.00)	0.31 (0.13, 0.73)	0.32 (0.13, 0.74)
SE	18 (1.4%)	0.82 (0.43, 1.58)	0.55 (0.27, 1.13)	0.66 (0.34, 1.29)
MW	9 (0.8%)	0.24 (0.08, 0.70)	0.28 (0.09, 0.86)	0.29 (0.10, 0.88)
SW	14 (1.9%)	1.15 (0.57, 2.30)	1.02 (0.50, 2.08)	1.03 (0.50, 2.10)
NW	13 (0.9%)	0.44 (0.21, 0.96)	0.36 (0.17, 0.78)	0.36 (0.17, 0.79)
CA	24 (0.6%)	Reference	Reference	Reference

*NE* Northeast, *SE* Southeast, *MW* Midwest, *SW* Southwest, *NW* Northwest, *CA* California

^a^61 observations had missing outcome data

^b^Adjusted for age, sex, income, and health insurance status

^c^Odds ratio for adjusted model with imputed data for missing values among covariates

### Sensitivity Analysis

There was a total of 53, 55, and 61 total observations that had missing data for the outcomes too expensive, language barriers, or transportation barriers, sequentially. There were three observations of missing data for health insurance status, four that were missing age data, and 206 that were missing hourly wage data. Sensitivity analysis resulted in adjusted OR estimates similar (within 0.11) to estimates found in the primary analysis.

## Discussion

We calculated the odds of specific barriers that cause farmworkers to have difficulty accessing health care in the U.S. Our study found regional variations in both the existence of barriers like cost, transportation, and language and demographic differences, including education, immigration, income, and health insurance status. Understanding regional disparities in the presence of barriers to accessing health care among farmworkers is an essential step to improving equitable health care access in the U.S. Variations between and within national regions may directly result from the nature of work within each region.

The overall OR for difficulty accessing health care was lower in the MW than all other regions for each potential barrier after adjusting for age, gender, income, and health insurance status. In the MW, farmworkers showed higher income, education, health insurance rates and were more likely to be born in the U.S. than farmworkers in all other regions. There were multiple patterns identified within the barriers to accessing health care. These patterns included lower odds of farmworkers employed in the MW reporting any of the barriers in this study compared to other regions. The disproportionate amount of MW farmworkers born in the U.S. compared to other regions could explain the lower odds of experiencing barriers. In line with previous research, English proficiency, health insurance status, education, and cultural differences influence health care access [20]. Being U.S.-born increases access to health insurance, higher wages, and education which in turn lessen barriers [21].

Farmworkers employed in the SE had greater difficulty accessing health care because of language barriers than farmworkers in other regions. Hoerster and colleagues found that individual-level factors, including gender, immigration status, English proficiency, transportation, and use of services outside the U.S., impacted farmworkers’ ability to access care [9]. In the U.S., speaking the English language increases the ability to identify and access health care services [22]. Unsurprisingly, when health care services are obtained, language barriers can result in negative perceptions of health care experiences and quality of care [22]. Language barriers not only affects health care disparities but also makes receiving preventative health information challenging [22].

Farmworkers employed in CA had difficulty accessing health care in the U.S. because it was too expensive or far away more than farmworkers in other regions. Not counting on health insurance causes concern to afford health care. In California, seven in ten adults are “somewhat” or “very” worried about their ability to afford care [23]. Many Californians rely on public programs such as Medicare and Medi-Cal (California’s Medicaid program) to support vulnerable populations. While the expansion of the Affordable Cares Act (ACA) helped increase enrollment many individuals still do not count on coverage [23]. On the positive end, California recently changed their Medi-Cal program to expand access to undocumented individuals, meaning it is likely many low-income farmworker families qualify for assistance through this modification [24]. It is possible that this change can help make health care services more affordable for vulnerable populations including farmworkers.

### Strengths

One strength of the study was using the NAWS dataset, implemented for over 30 years, providing a representative sample of farmworkers in the U.S. This study included nearly 10,000 participants, with the smallest region representing over 700 total participants. Only 274 (2.8%) participants were missing outcome or covariate data, and our sensitivity analysis suggested missing data was not a large factor in our results. Farmworkers are also an understudied population, and more studies are needed to improve the understanding of barriers farmworkers encounter when accessing health care [9].

### Limitations

This study focused on regional differences among farmworkers; however, challenges to accessing health care within each region may vary. Availability of state and county-level data could improve the understanding of geographical variations of barriers among farmworkers. While NAWS does use these variables during collection, employer and farm type data are not publicly available for analysis. Another limitation was that the collection relied on the self-reported outcomes and variable data from on-site interviews. The presence of other farmworkers or supervisors may influence survey responses. Additionally, the NAWS only collects data on farmworkers engaged in crop-related labor in the past year and does not represent farmworkers focused on other farm labor like mechanics or farmworkers who have been out of the labor force for over a year. Farmworkers also reported that they were not aware of barriers to accessing health care in the U.S. because they had never needed to access health care in the U.S. leading to concerns of underutilization of services that available to underserved communities. Lastly, the is limited research findings on differences of health care access by region. While much of the farmworker literature have common findings, there are not many findings that are specific to a region with the exception of California that is home to many farmworker-based research projects. Other states could benefit from their methodology to have precise needs for their farmworker communities which can help address health inequalities.

## Conclusion

A comprehensive strategy that incorporates individual and policy-level changes to expand access to health care for farmworkers should factor and tailor programs and interventions appropriate for farmworkers based on individual, community, state-level, and regional characteristics. For instance, other regions should look towards the MW and study the current policies in place that positively impact the low barriers to health care such as wages and employer-based health coverage. The SE could observe other region’s health care systems and include creative methods to help alleviate the language barrier between patients and providers, such as increasing the use of bilingual and bi-cultural community health workers. While California had higher reports of inability to afford health care cost, they are actively taking steps to attempt to reduce this barrier by modifying policies. Farmworkers who do not have health insurance and struggle to afford health care could be referred to Federally Qualified Health Centers (FQHCs) or Migrant Clinics. Yet, often vulnerable populations rely on emergency care as their main form of health care because they worry about the cost of routine care. Increased education on sliding fee scales can help bring awareness to a population who may not know of these local medical homes that are low cost and, in the long-run, more affordable than the emergency room. Furthermore, expanding access to affordable health care plans, mobile clinics, and other policies and programs that make the health system easier to navigate would help address barriers to accessing health care among farmworkers [9, 10]. Understanding regional disparities in the presence of barriers to accessing health care among farmworkers is an essential step to improving equitable access to care and highlight possible leads to issues that need to be addressed in their respective area.

## Abbreviations

NAWS: National agriculture workers survey
NE: Northeast
SE: Southeast
MW: Midwest
SW: Southwest
NW: Northwest
CA: California
